# The effectiveness of digital interventions for self-management of chronic pain in employment settings: a systematic review

**DOI:** 10.1093/bmb/ldae007

**Published:** 2024-07-07

**Authors:** Holly Blake, Wendy J Chaplin, Alisha Gupta

**Affiliations:** School of Health Sciences, Faculty of Medicine and Health Sciences, University of Nottingham, Queen’s Medical Centre, Derby Road, Nottingham, NG7 2HA, UK; NIHR Nottingham Biomedical Research Centre, Medical School, Queen’s Medical Centre, Derby Road, Nottingham, NG7 2UH, UK; School of Health Sciences, Faculty of Medicine and Health Sciences, University of Nottingham, Queen’s Medical Centre, Derby Road, Nottingham, NG7 2HA, UK; NIHR Nottingham Biomedical Research Centre, Medical School, Queen’s Medical Centre, Derby Road, Nottingham, NG7 2UH, UK; Population Health Sciences Institute, Baddiley-Clark Building, Newcastle University, Newcastleupon-Tyne, NE1 7RU, UK

**Keywords:** chronic pain, workplace, self-management, digital, occupational health, health promotion

## Abstract

**Introduction:**

Chronic pain affects over a quarter of the workforce with high economic burden for individuals, employers and healthcare services. Access to work-related advice for people with chronic pain is variable. This systematic review aims to explore the effectiveness of workplace-delivered digital interventions for the self-management of chronic pain.

**Source of data:**

MEDLINE, EMBASE, CINAHL, PsycINFO, the Cochrane Library, JBI, Open Science Framework, Epistemonikos and Google Scholar. Articles published between January 2001 and December 2023 were included. Searches were conducted between October 2023 and December 2023.

**Areas of agreement:**

Workplace-delivered digital interventions to support self-management of chronic pain at work may improve pain and health-related quality of life in vocationally active adults. Delivering interventions outside of clinical services, through the workplace setting, may help to reduce inequity in access to work-related advice for people with chronic pain, and ultimately reduce the burden on individuals, employers and healthcare services. Interventions include mobile apps and web-based programmes.

**Areas of controversy:**

Studies were moderate-to-low quality. Most studies focused on exercise, few considered other aspects of pain self-management. Given the limited evidence in the current literature, consensus on best intervention format and delivery is lacking.

**Growing points:**

More high-quality studies are needed given the heterogeneity in study design, interventions and outcome measures.

**Areas timely for developing research:**

No interventions included advice on work-related adjustments or support. Few studies included work-related outcomes, despite the known impact of pain on work and work on health.

## Introduction

Chronic pain is a global health priority. Prevalence estimates across 52 countries range from 9.9% to 50.3%^1^ with a high economic burden for individuals, employers and healthcare services (over £100 billion per annum in the UK^2^). Chronic pain can impact on people’s ability to work, their productivity, sickness absence, presenteeism and early retirement due to disability^3^^,^^4^. Retaining people with chronic pain in the workforce is important since unemployment is associated with an increased risk of mortality and morbidity, and good work improves health and wellbeing and reduces social exclusion.^5^ Providing advice and information to people with chronic pain about self-management strategies is recommended within clinical guidelines for chronic pain management.^6^ Self-management is equipping patients ‘with skills to actively participate and take responsibility in the management of their chronic condition in order to function optimally’ and may involve a combination of knowledge acquisition, sign/symptom monitoring, medication management, enhancing problem-solving/decision-making skills for medical treatment management and/or changing health behaviour(s).^7^ Self-management advice is routinely provided by healthcare professionals, but this rarely includes discussion about self-management strategies in the context of work. Although work-related self-management is a core focus of occupational therapy (OT), access to OT services is highly variable,^8^ meaning that many people with chronic pain do not receive work-related self-management advice. One route to supporting people to managing chronic pain at work (and potentially reducing burden on healthcare services) is to offer self-management interventions through employment settings. In the UK, around three-quarters of people aged 16–64 years are in employment. Given the high prevalence of chronic pain (one-third to one-half of the population), workplace-delivered interventions have potential for wide reach. Additionally, targeting interventions through non-clinical settings, such as workplaces, could help to reduce inequity in access to work-related advice and support through healthcare services. Digital interventions (DIs) are potentially scalable^9^ and may facilitate in reaching those with chronic pain regardless of their activity level, pain status, occupation type or geographical location. DIs provide information and/or support (emotional, decisional and/or behavioural) via digital platforms (e.g. website, computer, tablet or smartphone). Although workplace-focused DIs are emerging (e.g. Blake *et al*.^10^), the effectiveness of workplace delivered DIs in reducing pain, improving health, wellbeing, quality of life and work-related outcomes has not yet been established.

### Study aim

To conduct a systematic literature review to explore the effectiveness of DIs for self-management of chronic pain in employment settings.

## Methods

This systematic review was pre-registered with PROSPERO on October 19, 2023 (CRD42023463484).

### Eligibility criteria

All original studies consisting of randomized control trials (RCTs) and repeated measures non-randomized trials (RMs). The trials all included a DI and were conducted with vocationally active adult participants. The DI should function without any direct input from health professionals and require interaction with the participant. All studies were published since 2001, the year the term electronic health (eHealth) first emerged.^11^ Participants were recruited via their workplace. Articles were restricted to the English language, but there were no geographical limitations. Studies were excluded where the intervention was solely an appointment reminder or treatment compliance or telehealth or via email or direct input with a practitioner. Studies involving only passive monitoring (e.g. step counters only) or only reminders were excluded. Reviews, opinions, letters and unpublished literature were not considered.

### Search strategy

**Fig. 1:**
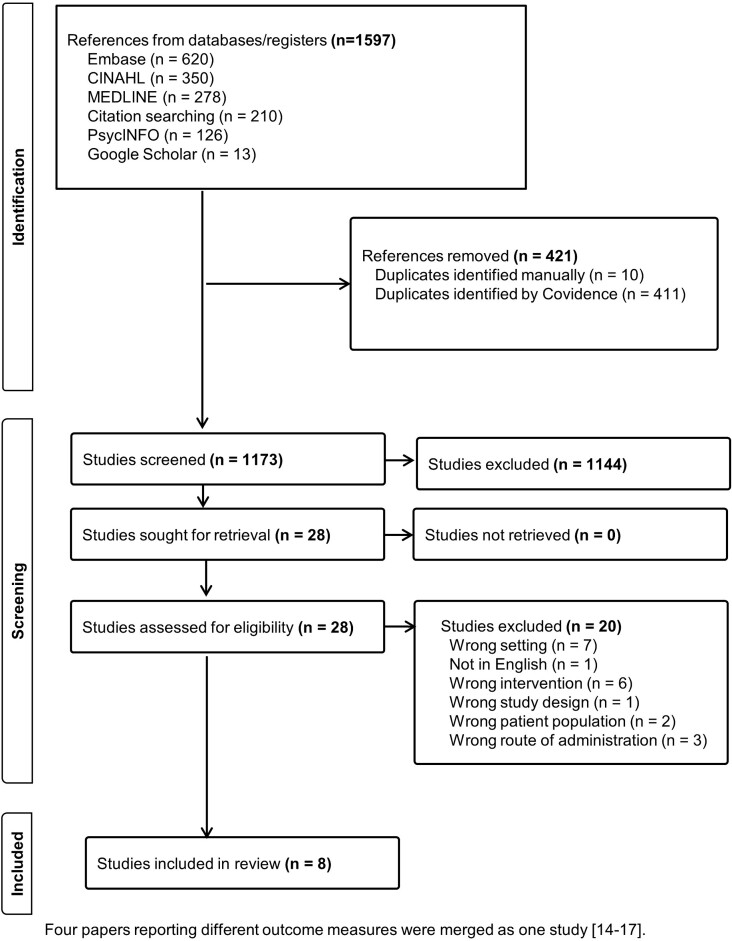
Flow chart showing article selection strategy, including reasons for exclusion according to the PRISMA guidelines.

This systematic review was conducted according to the Preferred Reporting Items for Systematic Reviews and Meta-Analyses: the 2020 PRISMA statement^12^ (Fig. 1). The following databases were searched electronically: MEDLINE, EMBASE, CINAHL, PsycINFO, the Cochrane Library, JBI, Open Science Framework, Epistemonikos and Google Scholar. Searches were conducted between October 2023 and December 2023. The search strategy (Supplementary File S1) was developed with a combination of Medical Subject Headings and keywords and using filters from the Cochrane Back Review Group. References of selected articles were hand-searched for eligible studies. A search of Open Grey and Google Scholar revealed materials with reference lists relevant to this review.

### Study selection

Two authors (W.J.C., H.B.) were involved in study selection. Records were managed through Covidence systematic review software (Veritas Health Innovation, Melbourne, Australia). An initial screening of titles and abstracts of studies retrieved was conducted (W.J.C.), to identify studies that meet the study inclusion criteria outlined above. A second reviewer (H.B.) independently screened 20% of titles and abstracts. Full text was obtained for abstracts with insufficient information or in a situation of disagreement. A study was included when both reviewers independently assessed it as satisfying the inclusion criteria from the full text. Any disagreements were resolved through discussion.

### Data items

Three authors (W.J.C., A.G. and H.B.) were involved in data extraction. Data extraction was independently performed on all included articles by two authors (W.J.C. and A.G.), a 20% check was conducted by a third author (H.B.). The following data were extracted: author and year, name of the journal, study design, inclusion/exclusion criteria, number of participants, participant characteristics (age, gender ratio), pain location, type of intervention, intervention duration, outcome measures (pain, quality of life, psychological, behavioural, physical activity, employment measures, other).

### Study risk of bias assessment

Risk of bias for each included trial was independently assessed by two reviewers (W.J.C. and A.G.) using the revised JBI critical appraisal tool for randomized controlled trials and the equivalent for quasi-experimental studies or non-randomized trials.^13^

### Strategy for data synthesis

We provide a narrative synthesis of the findings from the included studies, structured around study designs and settings, target population characteristics, type of intervention, intervention content and type of outcome. We provide summaries of intervention effects for each study by reporting between-group differences.

## Results

### Study selection

The search identified 1591 records and 28 full articles that met the inclusion criteria for further examination. Twenty articles were excluded. The findings of four articles were merged as they reported on a single study,^14–17^ and the remaining seven articles were selected for inclusion in the review. The articles were published between 2011 and 2021. The flowchart of the literature search is shown in Figure 1. Participant demographics and summary of interventions are shown in Table 1.

**Table 1:** Participant demographics and intervention type

Study # with ref.	Design	Partici pants *n*	Age, mean (SD)	Males *n* (%)	Digital intervention	Duration	Exercise/stretch	Breaks	Mind-fulness	Posture	Other
1^18^	RCT-pilot	41	C:41.7 (6.35), I: 40.48 (7.22)	C: 12 (29), I:7 (17.1)	Smartphone	6 weeks	✓	✓			
2^19^	RCT	121	C: 42.4 (8.7), I: 42.4 (8.0)	C:33 (72), I: 39 (81)	Smartphone AI-assisted chatbot	12 weeks	✓		✓	✓	Exercise motivation nudges
3^14–17^	RCT	100	C: 45.5 (7.02), I: 46.83 (9.13)	C:11 (4%), I:15.2%	Web-based	9 months	✓			✓	
4^20^	Cluster RCT	175	C:42.9 (12.0), I:41.8 (11.4)	C:77 (97.5), I: 85 (88.5	Computer-based	4 months	✓	✓			Education worksheets
5^21^	RCT-pilot	21	C:27.56 (4.67), I: 27.09 (4.83)	C:6 (55) I: 5 (45)	Smartphone	8 weeks	✓				
6^22^	Repeated measures	30	28.13 (2.97)	13 (66.50)	Smartphone	8 weeks	✓				
7^23^	Repeated measures	645	56 (12.83)	198 (30.7)	Web-based	6 months					Setting expectations, managing stress, coping with pain, accessing social support, healthy sleep, nutrition, exercise, improving doctor–patient relationships, medication adherence and chronic disease self-management.
8^24^	Repeated measures	417	49.8	128(30.7)	Web-based	30, 90, 180 days					Digital health coaching sources of stress, perceived barriers to managing stress, coping skills and resources and stages of change.

C, control; I, intervention web-based applications are accessed via the internet, computer-based applications were a software programme loaded onto an individual computer.

### Study designs and settings

The eight intervention studies^14–21^ included five RCTs, of which two were pilot RCTs^18^^,^^21^ and one was a cluster RCT,^20^ and three repeated measures (RMs) designs.^22–24^ Studies were conducted in six different countries, France =1,^20^ Spain =1,^14–17^ Japan = 1,^19^ Jordan =1,^18^ South Korea = 2,^21^^,^^22^ USA = 2.^23^^,^^24^ Most participants were office workers.

### Target population characteristics

A total of 1522 participants were included in the eight intervention studies. The number of participants in each study ranged from 20 to 645. Four interventions were conducted with university office workers^14^^,^^18^^,^^21^^,^^22^; the two RMs from USA included a small number of non-white collar workers.^23^^,^^24^ One study included workers who were manufacturing engineers of electronic components, but most were regarded as white-collar.^19^ The cluster RCT was based at two tyre factory research sites in four departments and included only office workers.^20^

### Type of interventions

Four studies involved smartphone mobile applications,^18^^,^^19^^,^^21^^,^^22^ one was computer-based software,^20^ and the remaining three were web-based.^14–17^^,^^23^^,^^24^ Six studies used interventions that included exercise.^14–22^ This usually took the form of stretching and mobilization and they were short (1–7 min duration). Of the exercise interventions, two studies included additional information to promote postural improvement,^14–17^^,^^19^ and two studies additionally encouraged frequent work breaks using computer ‘nudges’.^18^^,^^20^ One study included mindfulness and motivational nudges alongside the exercise intervention.^19^ Two further studies used digital health coaching that was designed to include education, stress management, psychological coping behaviours, and information to help people self-manage chronic pain.^23^^,^^24^ The duration of the interventions ranged from 6 weeks^18^ to 9 months.^14–17^

### Type of outcomes

All eight studies included at least one pain outcome measure, and most showed a statistically significant improvement in pain at follow-up. Five studies^16^^,^^18^^,^^21–23^ included a measure of health-related quality of life, and while different measurement instruments were employed, they all reported a significant improvement following the intervention. Seven studies^17^^,^^18^^,^^20–24^ included a psychological outcome measure, of which two studies reported improvements, one in work-related fear avoidance^21^ and one using the STarT Back Tool (SBST)—psychological subscale.^17^ Physical activity was assessed using the International Physical Activity Questionnaire^25^ in two studies.^18^^,^^20^ One study reported an improvement in readiness for physical activity using the Stages of Change questionnaire.^15^ One study^24^ reported a significant improvement in scores on the Work Productivity and Activity Impairment questionnaire,^26^ which measures impairments in both paid work (absenteeism and presenteeism) and unpaid work because of a health problem during the past 7 days. Other work-related metrics (such as stress at work and job satisfaction) were reported in one study,^20^ but there was no significant change. One study examined the acceptability of the DI^20^; another examined patient satisfaction and adherence rate.^22^ A summary of outcome variables is shown in Table 2.

**Table 2:** Outcome variables

**Study #** **lead author**	**Pain** **location**	**Inclusion**	**Exclusion**	**Pain**	**Quality of life**	**Psychological**	**Physical** **Activity**	**Behavioural**	**Employment metrics**	**Other**
1 Almhdawi ^18^	LBP	Aged 30–55 years, office worker >5y > 5 h daily desk work. SR LBP > 3 months + 3/10 VAS. Smartphone	Pregnancy, specific back diagnosis	**VAS** B: 5.62 ± 2.06 FU: 2.30 ± 2.13, *P* < 0.001 **ODI** B:30.95 ± 9.31 FU: 20.25 ± 13.47, *P* = 0.002 Cohen’s d = 1.71	SF-12: **PCS,** MCS B: 67.67 ± 17.64 F:79.95 ± 16.09 *P* = 0.001 PCS Cohen’s d = 1.08 MCS Cohen’s d = 0.131	DASS Cohen’s d Stress =0.34, Cohen’s d Anxiety =0.18 Cohen’s d Depression 0.20	IPAQ Cohen’s d = 0.45		Hours, days, sickness, mean computer time, phone usage,	PSQI Cohen’s d = 0.14
2 Anan ^19^	Neck, shoulder and back	Screening questions smartphone	Pregnancy, CVD, other clinical trials, disability/exercise restriction	**Subjective pain severity neck/shoulder and back + subjective pain stiffness neck/shoulder and back + improvement** 36 (76%) improved *P* < 0.001						Adherence rate 92% (44/48)
3 del Pozo-Cruz ^14–17^ (four articles, 1 study)	Back	18–64 years, + diagnosis of subacute LBP physical activity < 2 working > 6 h on a computer daily	Diagnosed backache, reported chronic backache, clinical red flags for disc disease, any other major illness, lack of fluency in Spanish	**ODI,** OR 5.40 (95% CI 1.71, 17.22), *P* = 0.001 Cohen’s d = 0.93 **RDM** B:12.28 ± 2.63 F: 4.93 ± 2.59, *P* < 0.001 Cohen’s d = 2.82 **SBST** (TOTAL) B: 4.38 (1.48) F:3.39 (1.39), *P* = 0.019 Cohen’s d = 0.98	**EQ-5D-3L** OR 3.59 (95% CI 2.21, 5.82), *P* < 0.001 Cohen’s d = 0.71	**SBST psych** **Fear avoidance.** OR 0.35 (95% CI (0.15, 0.84), *P* = 0.017 Cohen’s d = 0.58	**Lumbar endurance** B: 78.80 ± 30.6 F: 92.4 ± 27.9 *P* < 0.001 Cohen’s d = 0.46 **Abdominal endurance** B: 48.1 ± 33.0 F:64.4 ± 30.7, *P* < 0.001 Cohen’s d = 0.51	**Stage of change questionnaire**		
4 Lanhers ^20^	Any	18–65 years + > 5 h VDU per day + no sickness absence in 1 month + admin staff no behavioural or learning disorder + no new VDU equipment	Maternity leave, change in job	**Nordic questionnaire** Reduction in 30 days, *P* = 0.038		HADS	IPAQ sedentary time per day		Stress at work, satisfaction at work,	DI acceptability, eye strain, corrective spectacles or lenses, exercise adherence
5 Lee ^21^	Neck	25–35 years 6 + computer hours neck pain for more than 6 months	<3 VAS pain A history of traumatic injury on neck, a congenital deformity, a history of surgical operation or injection on neck and any neurological symptoms.	**VAS,** B:5.20 ± 2.19 F:2.73 ± 1.99, *P* < 0.05 pain duration, McKenzie classification, Cohen’s d = 1.18 **Functional disability**—**neck disability index questionnaire, strength** and ROM B: 26.80 ± 9.68 F:17.25 ± 8.34, *P* < 0.05 Cohen’s d = 1.06	**SF-36** **PCS,** B:43.18 ± 8.58 F: 48.40 ± 7.22, *P* < 0.05 Cohen’s d = 0.67	**Fear-avoidance (Work)** B: 25.18 ± 3.97 F:20.73 ± 6.4 *P* < 0.05 Cohen’s d = 0.84				**Maximal voluntary strength** Neck extension B:16.82 ± 7.74 F:25.92 ± 6.86, *P* < 0.05 Cohen’s d = 1.24
6 Lee^22^	Neck	Neck pain > 3 months; mean pain > 3 in the last week; smartphone	(1) They had received any other treatment or surgery within 3 months; or (2) Their neck pain was caused by a known trauma, rheumatic disorder or malignant disease.	**VAS**, B:4.63 ± 1.89 F:2.0 ± 1.69, *P* < 0.001 Cohen’s d = 1.47 pain duration, McKenzie classification, **Functional disability—Neck disability index questionnaire, strength** and ROM B:22.18 ± 9.42 F:13.74 ± 7.28, *P* < 0.001 Cohen’s d = 1.00.	**SF-36** **PCS,** B: 46.14 ± 7.29 F:51.22 ± 6.55, *P* = 0.02 Cohen’s d = 0.73	Fear-avoidance. (Work)	Exercise minutes per day and sedentary minutes per day		Hours, days, mean computer time,	The patient satisfaction was 3.91 ± 0.51/5 The adherence rate was 91.85% (37.78%), and the mean duration per exercise session was 16.8 ± 7.38 min
7 Nevedal ^23^	Any	Eligible participants were either employed by 1/37 participating US companies or a member of 1 of 18 participating US healthcare plans.	This program isn’t intended for people suffering from the following: acute pain, cancer pain, pelvic or abdominal pain.	**VAS**, B:5.30 ± 2.46 F:3.72 ± 2.73, *P* < 0.001 Cohen’s d = 0.61 duration, McKenzie classification, Functional disability, neck pain, pain interference, **pain unpleasantness**, pain medication B:5.43 ± 2.52 F:3.78 ± 279, *P* < 0.001 Cohen’s d = 0.62	**Quality of life—CDC HRQOL-4 (1 item)** B: <Fair 20.6% F: <Fair 16.5%, *P* = 0.006	Participant stress		Motivation to manage and confidence to manage pain		Quality of health—CDC HRQOL-4 (1 item)
8 Silberman ^24^	Any	First, subjects must have participated in: HealthMediaR CareTM for pain (a chronic pain management program);		**Pain improvement and pain worsening were both associated with productivity impairment.** *P* < 0.001		CES-D Boston Form Depression/10			**Work productivity and activity impairment questionnaire** B:37.62 F: 29.29, *P* < 0.001 Cohen’s d unavailable	

Only significant findings are reported. **BOLD** items showed statistically significant change.

VAS, Visual Analogue Scale; ODI, Oswestry Disability Index; RDM, Roland-Morris Disability Questionnaire; SBST, STarT Back Tool; CES-D, Center for Epidemiologic Studies Compression Scale; SF-36, Short Form Survey, SF-12 Short Form Survey, PCS, Physical Component Score (SF-12), DASS, Depression Anxiety Stress Scale; ROM, Range of Movement; HADS, Hospital Anxiety and Depression Score; PSQI, Pittsburgh Sleep Quality Index; VDU, visual display unit; IPAQ International Physical Activity Questionnaire; Quality of life/health-CDC HRQOL-4, Centers for Disease Control and Prevention Health-related Quality of Life 4 item Measure; B, baseline; F, follow-up; OR, odds ratios.

### Risk of bias

All the included studies were independently assessed for risk of bias by two reviewers (W.J.C. and A.G.) with an initial agreement of 86.96%. Disagreements were resolved by discussion between the reviewers to reach a consensus. The JBI Critical Appraisal Tool for RCTs^13^ and Quasi Experimental Studies^27^ for the RMS was followed (Supplementary File S2). In common with many behavioural interventions, blinding was not included in many studies and the design of RM’s did not permit comparison. Two studies had low risk of bias,^14^^,^^18^ the remainder were moderate risk.

## Discussion

This systematic review shows that workplace-delivered DIs to support self-management of chronic pain at work may improve pain and health-related quality of life in vocationally active adults. Delivering interventions outside of clinical services, through the workplace setting, may therefore help to reduce inequity in access to work-related advice for people with chronic pain, and ultimately reduce the burden on individuals, employers and healthcare services. Interventions in this review were delivered using mobile applications (mHealth), computer-based or web-based approaches (e-Health). mHealth and eHealth are becoming increasingly popular for self-management of chronic pain and have positive outcomes for reducing pain intensity and improving quality of life and functional disability in a range of chronic pain conditions (e.g. see literature^28–30^). Given the limited evidence published to date on workplace-delivered interventions, it is difficult to draw conclusions from this review on the most appropriate intervention format, delivery mode or long-term effects.

A limitation of the review is that the included studies showed a high degree of heterogeneity in study design, interventions and outcome measures, so a meta-analysis could not be conducted. We found no published evidence for interventions delivered in UK employment settings. Our review demonstrates that DIs delivered in the workplace largely focus on exercise, with few studies (if any) considering other aspects of chronic pain self-management (e.g. psychological support, behavioural strategies, health behaviours). Factors specific to the workplace (e.g. disability disclosure, reasonable adjustments from the employer) are rarely included in workplace delivered DIs for self-management of chronic pain, despite the known impacts of chronic pain on work^31^ and work on health.^5^ Similarly, outcomes measured in included studies were largely related to pain and health-related quality of life, with few considering psychological factors known to influence pain self-management and the self-perceived burden of chronic pain, such as self-efficacy, anxiety and depression.^32^^,^^33^

Although the interventions in our included studies were delivered via the workplace, studies rarely measured outcomes specific to the workplace and employment (e.g. absenteeism, presenteeism, work capacity, job-related factors, etc.). This concurs with findings from a prior systematic review showing that few studies report on work-related impacts, strategies or outcomes.^34^ Emerging research will address this gap in the evidence and consider the broader aspects of self-management such as barriers to work, facilitators of work ability, workplace pain self-management strategies, as well as measuring a broader range of health, wellbeing and work-related outcomes.^35^

This review has implications for employers and healthcare services. Workforce health and wellbeing is increasingly recognized as a key component of business performance and corporate social responsibility. Employers should therefore consider incorporating evidence-based DIs to support vocationally active adults with the management of chronic pain at work within workforce health and wellbeing and/or occupational health provisions. Increasing research in this field will help with future recommendations to employers about the *types* of DIs that would be most beneficial for workers (e.g. health and wellbeing) and to organizational outcomes (e.g. sickness absence and indices of business performance). Digital self-management interventions for chronic pain have potential to reduce burden on healthcare services by increasing access to self-management advice and support outside of clinical settings. These interventions provide, for example, an additional source of assistance for individuals who are not accessing healthcare services or are on waiting lists for work-related advice and support (i.e. OT).

## Author contributions

Holly Blake (Conceptualization, Methodology, Formal Analysis, Investigation, Validation, Visualization, Supervision, Writing—original draft), Wendy J. Chaplin (Data curation, Methodology, Formal Analysis, Investigation, Methodology, Project administration, Software, Validation, Visualization, Writing—original draft), and Alisha Gupta (Formal Analysis, Writing—review and editing)

## Conflict of interest statement

The authors declare that they have no conflict of interest.

## Funding

No external source of funding was used.

## Data availability

The data underlying this article are available in the article and in its online Supplementary Material. No new data were generated or analysed in support of this review.

## Ethical approval

This article does not contain any studies with human participants or animals performed by any of the authors.

## Informed consent

For this type of study, informed consent is not required.

## Supplementary Material

Supplementary_file_S1_ldae007

Supplementary_file_S21_ldae007
