# Supplementary material for: The effectiveness of digital interventions for self-management of chronic pain in employment settings: a systematic review
Source: Br Med Bull. 2024 Jul 7;151(1):36–48. doi: 10.1093/bmb/ldae007 (PMC11436954; doi:10.1093/bmb/ldae007)
Supplement: Supplementary_file_S1_ldae007 [file supplementary_file_s1_ldae007.docx]

**Supplementary file S1: MEDLINE (OVID) Search strategy**

Title: Digital and pain interventions at work: systematic review

- PAIN

1. Pain/
2. Chronic Pain/
3. exp Musculoskeletal Pain/
4. exp Complex Regional Pain Syndromes/
5. Fibromyalgia/
6. exp Abdominal Pain/
7. exp Ehlers-Danlos Syndrome/
8. exp Cumulative Trauma Disorders/
9. exp Elbow Tendinopathy/
10. exp Arthritis/
11. exp Hyperostosis/
12. exp Neuralgia/
13. exp Headache/
14. exp Chest Pain/
15. exp Back Pain/ or Shoulder Pain/ or Neck Pain/
16. exp Pelvic Pain/
17. exp Migraine Disorders/
18. (pain* adj3 (chronic or persist* or musculo-skeletal or musculoskeletal or arthrit*)).mp.
19. (pain* adj1 (sever* or intens* or interfer*)).mp.
20. (pain* adj1 (abdominal* or gastro-intestin* or gastrointestin* or angina* or migraine* or headache* or gynae* or pelvi* or chest or back or neck or shoulder)).mp.
21. (headache* or neuralg* or dysmenorr* or arthritis).mp.
22. (pain* adj4 Crohn*).mp.
23. (pain* adj4 ("degenerative disc disease*" or "degenerative disk disease*")).mp.
24. Intervertebral Disc Degeneration/
25. (fibromyal* or fibrositis or fibromyositis or FMS).mp.
26. "tennis elbow*".mp.
27. "ehlers danlos".mp.
28. "repetit* strain injur*".mp.
29. Hyperostosis, Diffuse Idiopathic Skeletal/
30. "diffuse idiopathic skeletal hyperostosis".mp.
31. exp Complex Regional Pain Syndromes/
32. ((chronic or complex) adj "regional pain syndrome*").mp.
33. CRPS.mp.
34. 1 or 2 or 3 or 4 or 5 or 6 or 7 or 8 or 9 or 10 or 11 or 12 or 13 or 14 or 15 or 16 or 17 or 18 or 19 or 20 or 21 or 22 or 23 or 24 or 25 or 26 or 27 or 28 or 29 or 30 or 31 or 32 or 33

- WORKPLACE

1. exp Workplace/
2. (workplace* or worksite*).mp.
3. ((work* or employ*) adj3 (place* or site* or setting* or environment* or office* or factory or factories or desk or sedentary)).mp.
4. 35 or 36 or 37

- DIGITAL INTERVENTIONS

1. exp Telemedicine/
2. Digital Technology/
3. Mobile Applications/
4. Internet-Based Intervention/
5. exp Computers, Handheld/
6. Internet/
7. (digital adj (tech* or intervention*)).mp.
8. ("virtual medicine" or tele-medicine or telemedicine or tele-rehab* or telerehab* or "mobile health" or tele-health or telehealth or ehealth or e-health or mhealth or m-health).mp.
9. ((mobile or smart or smartphone or smart-phone) adj (app or apps or application* or device*)).mp.
10. (computer* adj1 (handheld or tablet*)).mp.
11. (internet or internet-based or internet-enabled or online or on-line or web or web-based or web-mediated or app-based or technology-based).mp.
12. (e-health or ehealth).mp.
13. 39 or 40 or 41 or 42 or 43 or 44 or 45 or 46 or 47 or 48 or 49 or 50
14. 34 and 38 and 51
15. limit 52 to yr="2001 -Current"
