# Supplementary material for: The effectiveness of digital interventions for self-management of chronic pain in employment settings: a systematic review
Source: Br Med Bull. 2024 Jul 7;151(1):36–48. doi: 10.1093/bmb/ldae007 (PMC11436954; doi:10.1093/bmb/ldae007)
Supplement: Supplementary_file_S21_ldae007 [file supplementary_file_s21_ldae007.docx]

**Supplementary File S2**

**Table A – JBI Critical appraisal checklist for randomized controlled trials**

Reviewer Date

Author Year Record Number

Yes No Unclear NA

1. Was true randomization used for assignment of
participants to treatment groups? □ □ □ □

2. Was allocation to treatment groups concealed? □ □ □ □

3. Were treatment groups similar at the baseline? □ □ □ □

4. Were participants blind to treatment assignment? □ □ □ □

5. Were those delivering treatment blind to
treatment assignment? □ □ □ □

6. Were outcomes assessors blind to treatment
assignment? □ □ □ □

7. Were treatment groups treated identically other
than the intervention of interest? □ □ □ □

8. Was follow up complete and if not, were differences
between groups in terms of their follow up adequately
described and analyzed? □ □ □ □

9. Were participants analyzed in the groups to which they
were randomized? □ □ □ □

10. Were outcomes measured in the same way for
treatment groups? □ □ □ □

11. Were outcomes measured in a reliable way? □ □ □ □

12. Was appropriate statistical analysis used? □ □ □ □

13. Was the trial design appropriate, and any deviations from
the standard RCT design (individual randomization, parallel
groups) accounted for in the conduct and analysis of the trial? □ □ □ □

Overall appraisal:
Include □Exclude □ Seek further info □

Comments (Including reason for exclusion)

**Table B – JBI Critical appraisal checklist for quasi-experimental studies (non-randomized experimental studies)**

Reviewer Date

Author Year Record Number

Yes No Unclear NA

14. Is it clear in the study what is the ‘cause’ and what is the ‘effect’ (i.e. there is no confusion about which variable comes first)? □ □ □ □

15. Were the participants included in any comparisons similar? □ □ □ □

16. Were the participants included in any comparisons receiving similar treatment/care, other than the exposure or intervention of interest? □ □ □ □

17. Was there a control group? □ □ □ □

18. Were there multiple measurements of the outcome both pre and post the intervention/exposure? □ □ □ □

19. Was follow up complete and if not, were differences between groups in terms of their follow up adequately described and analyzed? □ □ □ □

20. Were the outcomes of participants included in any comparisons measured in the same way? □ □ □ □

21. Were outcomes measured in a reliable way? □ □ □ □

22. Was appropriate statistical analysis used? □ □ □ □

Overall appraisal:
Include □Exclude □ Seek further info □

Comments (Including reason for exclusion)

**Table C - JBI Critical Appraisal Checklist Results**

|  | Almhdawi, 2020 | Anan, 2021 | Del Pozo Cruz, 2012, | Lanhers, 2016 | Lee, 2017 | Lee,2017 | Nevedal, 2013 | Silberman, 2011 |
| --- | --- | --- | --- | --- | --- | --- | --- | --- |
| Rank (High, Medium, Low) | **L** | **M** | **L** | **M** | **M** | **M** | **M** | **M** |
| Question number | |  |  |  |  |  |  |  |
| **RCT Studies** |  |  |  |  |  |  |  |  |
| 1 | Y | Y | Y | N/A | Y |  |  |  |
| 2 | Y | Y | Y | Y | Y |  |  |  |
| 3 | Y | N/A | Y | N | Y |  |  |  |
| 4 | Y | N | N | N | N |  |  |  |
| 5 | Y | N/A | N/A | N/A | ? |  |  |  |
| 6 | Y | Y | Y | Y | Y |  |  |  |
| 7 | Y | N | Y | Y | ? |  |  |  |
| 8 | Y | Y | Y | Y | Y |  |  |  |
| 9 | Y | Y | Y | Y | Y |  |  |  |
| 10 | Y | Y | Y | Y | Y |  |  |  |
| 11 | Y | Y | Y | Y | Y |  |  |  |
| 12 | Y | Y | Y | Y | Y |  |  |  |
| 13 | Y | Y | Y | Y | Y |  |  |  |
| **Quasi-Experimental** |  |  |  |  |  |  |  |  |
| 14 |  |  |  |  |  | Y | Y | Y |
| 15 |  |  |  |  |  | N/A | N/A | N/A |
| 16 |  |  |  |  |  | N/A | N/A | N/A |
| 17 |  |  |  |  |  | N | N | N |
| 18 |  |  |  |  |  | Y | Y | Y |
| 19 |  |  |  |  |  | Y | Y | N |
| 20 |  |  |  |  |  | Y | Y | N |
| 21 |  |  |  |  |  | Y | Y | Y |
| 22 |  |  |  |  |  | Y | Y | Y |

Y = yes; N = no; ? = unclear; N/A = not applicable

Risk of bias thresholds: RCT: Low 13-12; Moderate 10-9; High ≤8

Quasi-Experimental: Low 8, 9; Moderate 7, 6; High <6
